# Differential Effects of Brain Death and Circulatory Death on Myocardial Integrity and Transplant Outcomes

**DOI:** 10.26502/jsr.10020466

**Published:** 2025-08-20

**Authors:** Chang Kon Kim, Shaanali Mukadam, Devendra K Agrawal

**Affiliations:** Department of Translational Research, College of Osteopathic Medicine of the Pacific, Western University of Health Sciences, Pomona, California 91766, USA

**Keywords:** Brain death, Cardiac allograft vasculopathy, Circulatory death, Donation after circulatory death, Donor brain death, Donor-derived cell-free DNA, Heart transplantation, Heterotopic heart transplantation, Immunologic rejection, Ischemia-reperfusion injury, Primary graft dysfunction

## Abstract

Heart transplantation is the definitive treatment for end-stage heart failure, yet the persistent scarcity of donor organs has necessitated expanded criteria for donor selection, particularly the inclusion of donors after brain death (DBD) and circulatory death (DCD). These two mechanisms of donor death result in distinct pathophysiological alterations that impact myocardial viability, inflammatory activation, and immune recognition. DBD is characterized by a catecholamine surge, hormonal collapse, and systemic inflammation, contributing to endothelial dysfunction and immunologic priming. In contrast, DCD grafts are subjected to warm ischemia and reperfusion injury, elevating the risk of primary graft dysfunction and delayed recovery. These physiological differences may differentially influence graft performance, immunologic rejection, infection risk, and long-term survival. This review presents a detailed analysis of how the cause of donor death influences clinical outcomes in heart transplantation. It explores the mechanistic underpinnings of DBD- and DCD-associated injury, assesses their impact on post-transplant complications, and evaluates emerging strategies such as ex vivo perfusion, donor-derived cell-free DNA monitoring, and gene expression profiling. Additionally, it discusses how donor physiology intersects with recipient characteristics, the selective use of heterotopic transplantation, and evolving approaches in immunosuppression and risk stratification. These insights support the development of precision-guided protocols that integrate donor and recipient profiles to optimize graft utilization and improve outcomes.

## Introduction

Heart failure remains a leading cause of morbidity and mortality worldwide, with advanced-stage disease affecting millions and contributing substantially to healthcare costs and patient burden [1]. For individuals with end-stage heart failure who are refractory to medical and device-based therapies, heart transplantation remains the gold standard for extending survival and improving quality of life [2]. However, the persistent shortage of suitable donor hearts has necessitated expanding the criteria for acceptable donors, bringing increasing attention to the physiological condition of the donor at the time of death.

Among the most critical determinants of graft viability is the donor’s mechanism of death, typically classified as either donation after brain death (DBD) or donation after circulatory death (DCD) [3]. These two categories differ not only in the clinical protocols surrounding organ retrieval but also in the cascade of physiological, hormonal, and immunological events that precede procurement. Brain death is associated with a sudden and profound neurogenic catecholamine surge, leading to systemic inflammation, hemodynamic instability, and myocardial stress [4]. In contrast, DCD involves a period of warm ischemia following cardiac arrest and before organ perfusion, raising concerns about ischemia-reperfusion injury and delayed graft function [5].

Emerging studies suggest that these distinct donor conditions may have significant implications for short- and long-term transplant outcomes, including primary graft dysfunction, immunologic rejection, infection risk, and the development of chronic complications such as cardiac allograft vasculopathy [6]. Furthermore, as technologies such as normothermic regional perfusion and ex vivo perfusion platforms evolve, the clinical viability of DCD hearts is being redefined, necessitating a deeper understanding of the biological differences between donor types [7].

### Donor Categories and Pathophysiological Considerations

The physiological state of the donor at the time of organ procurement plays a pivotal role in determining the immediate and long-term viability of the transplanted heart [8]. Donors are typically classified into two primary categories based on the mechanism of death: donation after brain death (DBD) and donation after circulatory death (DCD) [6]. While both sources can yield transplantable organs, each is associated with distinct pathophysiological processes that may differentially influence graft quality and post-transplant outcomes.

### Donation After Brain Death

**Figure 1:**
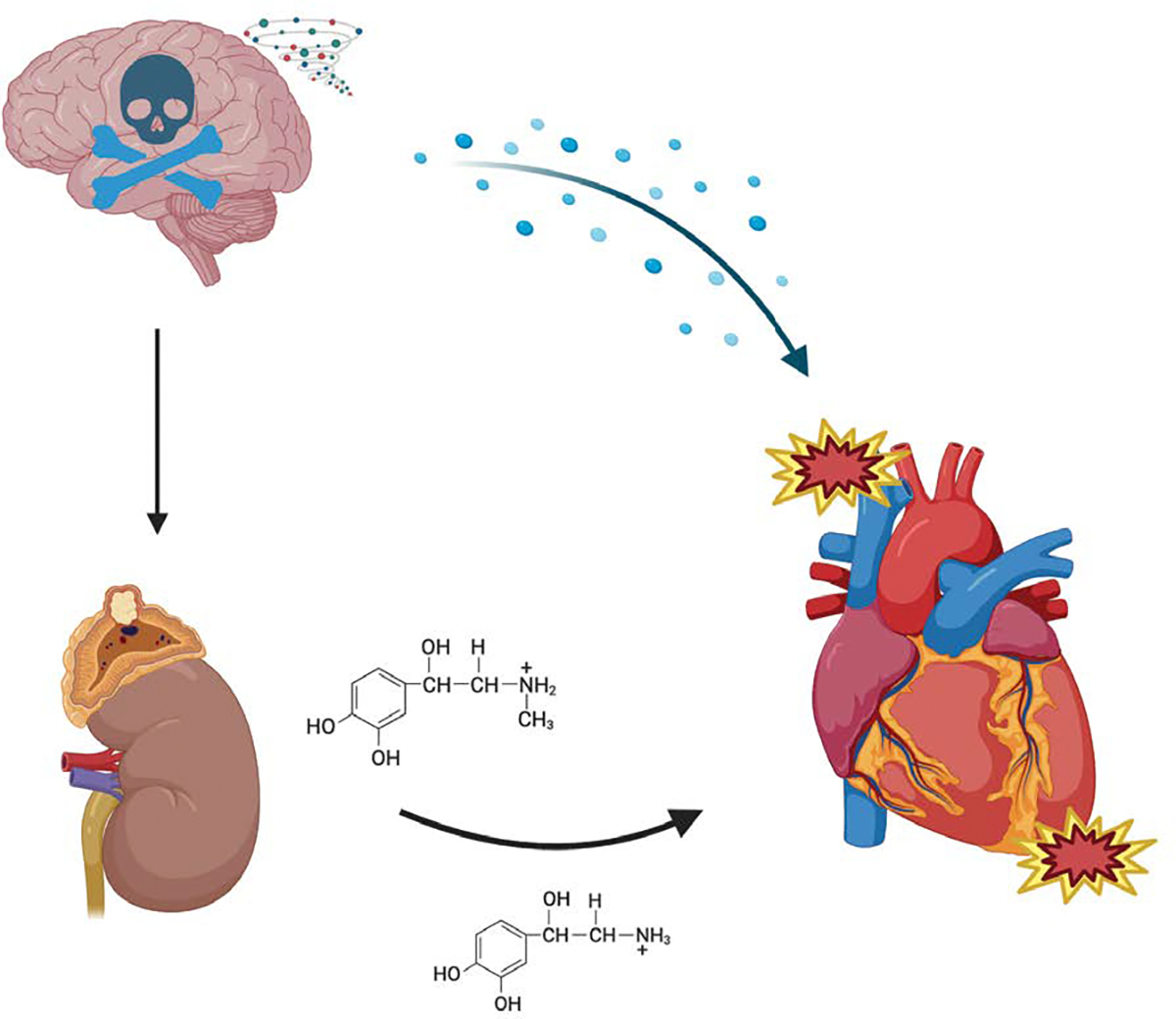
Brain death triggers a massive release of catecholamines due to hypothalamic dysfunction and rising intracranial pressure. This surge causes hemodynamic instability and contributes to myocardial injury, ischemia, and structural cardiac damage, potentially compromising donor heart quality.

Brain death results from catastrophic intracranial events such as trauma, hemorrhage, or stroke, leading to complete and irreversible loss of brain function [3]. The ensuing physiological response is characterized by a “catecholamine storm,” a massive surge of endogenous epinephrine and norepinephrine secondary to increased intracranial pressure and hypothalamic dysfunction [4] (Figure 1). This acute neurogenic stress induces profound hemodynamic instability, including tachycardia and hypertension, followed by peripheral vasoconstriction. Although initially compensatory, this surge can result in myocardial injury, subendocardial ischemia, and cardiomyocyte apoptosis.

Hormonal dysregulation is another hallmark of donation after brain death (DBD) [9]. The loss of hypothalamic-pituitary axis function leads to hypothyroidism, relative adrenal insufficiency, and antidiuretic hormone deficiency, each of which may impair myocardial performance and electrolyte homeostasis. To mitigate these effects, some centers employ hormonal resuscitation protocols, including corticosteroids, vasopressin, and thyroid hormone supplementation, with varying degrees of success.

### Donation After Circulatory Death

**Figure 2:**
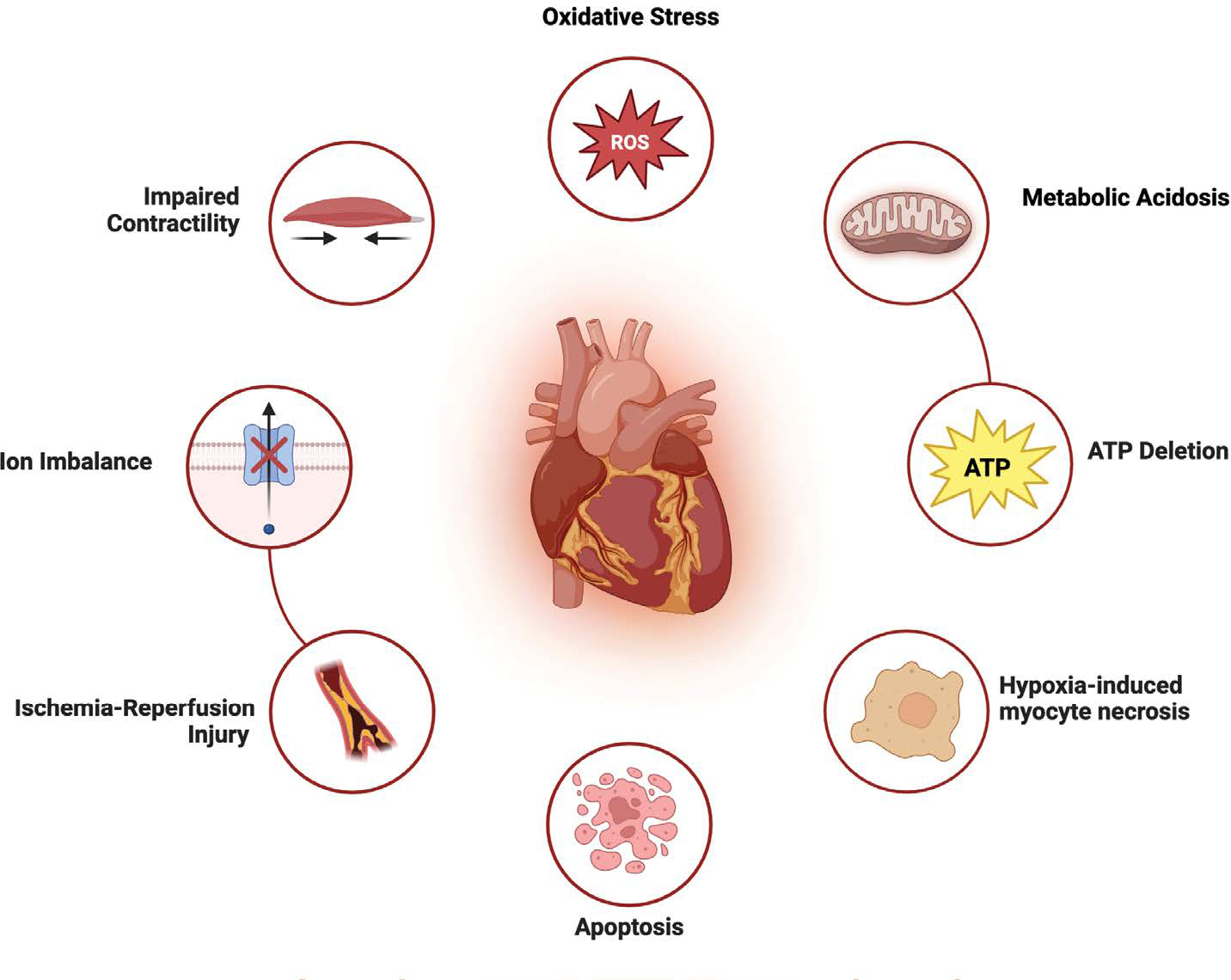
In donation after circulatory death (DCD), the period of warm ischemia following cardiac arrest leads to global myocardial hypoxia. This triggers a cascade of metabolic and structural injuries—including oxidative stress, ATP depletion, ion imbalance, and mitochondrial dysfunction—that contribute to primary graft dysfunction and impaired contractility.

In donation after circulatory death (DCD), the heart is procured following cardiac arrest and cessation of circulatory function, typically after withdrawal of life-sustaining therapy [5]. Unlike DBD, where organ perfusion is maintained until procurement, DCD is characterized by a warm ischemia period between the cessation of circulation and initiation of organ preservation measures (Figure 2). This period of global myocardial hypoxia can result in metabolic acidosis, ATP depletion, and ischemia-reperfusion injury upon reperfusion, all of which contribute to primary graft dysfunction (PGD) and may impair long-term graft integrity [10].

Although DCD donors generally have lower circulating inflammatory markers compared to DBD donors, the period of ischemia introduces other types of injury that affect mitochondrial integrity, ion homeostasis, and myocyte survival [11]. Despite these challenges, early data suggest that with proper preservation strategies, DCD hearts can achieve comparable outcomes to DBD hearts in select recipients [6].

### Early Post-Transplant Outcomes and Donor Cause of Death

The early postoperative period following heart transplantation is a critical window in which graft viability, host adaptation, and the foundation for long-term survival are established. Among the most significant early complications is primary graft dysfunction (PGD), a syndrome of acute allograft failure that occurs in the absence of rejection or surgical complications [12]. The risk and severity of PGD are closely linked to the mechanism of donor death, with both donation after brain death (DBD) and donation after circulatory death (DCD) imposing unique pathophysiological stressors on the myocardium [13].

### Primary Graft Dysfunction

Primary graft dysfunction (PGD) is defined by the severe impairment of graft function within the first 24 to 72 hours after transplantation, affecting either the left ventricle, the right ventricle, or both [14]. It is characterized by low cardiac output, hypotension, elevated filling pressures, and poor response to inotropic support, often necessitating mechanical circulatory support such as venoarterial extracorporeal membrane oxygenation (VA-ECMO). The severity is commonly graded using the International Society for Heart and Lung Transplantation (ISHLT) classification, which categorizes PGD as mild, moderate, or severe based on hemodynamic criteria and the need for mechanical support.

### Primary Graft Dysfunction in Donation After Circulatory Death

In DCD transplantation, the heart is procured only after the irreversible cessation of circulatory function following the planned withdrawal of life-sustaining therapy [15]. This inherently introduces a period of warm ischemia—a time during which the heart is not being perfused but remains at body temperature—posing a substantial risk for ischemia-reperfusion injury.

A critical metric in this context is the functional warm ischemic time (FWIT), defined as the interval between the onset of sustained systolic hypotension (often MAP <50 mmHg or loss of pulse pressure) and the initiation of graft preservation [16]. During this period, myocardial oxygen delivery ceases, leading to ATP depletion, mitochondrial dysfunction, calcium overload, and the generation of reactive oxygen species (ROS) upon reperfusion. The extent of this injury is directly proportional to the duration of FWIT, and studies suggest that exceeding 30 minutes significantly increases the risk of severe PGD and early graft failure [17].

Normothermic regional perfusion (NRP) is a technique that restores oxygenated blood flow to the donor heart in situ after circulatory death. Ex vivo perfusion platforms have shown promise in mitigating ischemic injury by reconditioning the graft prior to implantation. However, despite these advances, DCD hearts remain at increased risk of PGD, especially in cases where warm ischemia is prolonged or NRP cannot be performed [6]. Clinical studies have demonstrated higher rates of early mechanical circulatory support in DCD recipients, although mid-term survival appears comparable to DBD recipients when modern preservation techniques are employed.

### Primary Graft Dysfunction in Donation After Brain Death

In contrast to DCD, DBD donors maintain circulation until the point of organ procurement. However, brain death initiates a distinct and equally injurious cascade of physiological derangements. The progression to brain death typically involves a rapid increase in intracranial pressure, which leads to Cushing’s reflex (hypertension, bradycardia, irregular respiration), followed by brainstem herniation and the loss of autonomic regulation [18]. The initial phase is marked by a “catecholamine storm,” during which massive sympathetic discharge causes severe vasoconstriction, tachycardia, and a surge in myocardial oxygen demand. This may precipitate subendocardial ischemia, contraction band necrosis, and ventricular arrhythmias.

As brainstem function ceases, the sympathetic surge collapses into hypotension, bradycardia, and vasoplegia, compromising coronary perfusion and precipitating myocardial stunning. Myocardial stunning in this context refers to transient but reversible systolic dysfunction due to metabolic and adrenergic injury rather than permanent structural damage [19]. These changes are compounded by inflammatory cytokine release (e.g., TNF-α, IL-6, IL-1β), leukocyte infiltration, and complement activation—all of which contribute to endothelial dysfunction and myocardial edema.

DBD-associated PGD tends to present with diffuse biventricular dysfunction, often accompanied by diastolic impairment and elevated filling pressures, particularly if aggressive fluid resuscitation was required pre-procurement. However, because the injury is more often functional and inflammatory, rather than necrotic, these grafts may respond better to pharmacologic inotropes and afterload reduction, and in some cases, recover fully without mechanical support.

### Perioperative Complications

The perioperative period of heart transplantation is a highly dynamic phase in which the physiological integrity of the donor heart is tested under the stress of procurement, transport, implantation, and reperfusion [4]. Donor physiology, particularly as shaped by the mechanism of death, significantly influences how the graft tolerates this process and determines the recipient’s immediate postoperative course [8].

In donors after brain death, the heart is typically exposed to prolonged periods of systemic instability prior to procurement. The progression from elevated intracranial pressure to brainstem herniation is accompanied by a powerful, transient sympathetic surge that markedly increases myocardial oxygen demand. This initial “catecholamine storm” often results in subendocardial ischemia and myocyte calcium overload [4]. Following brainstem death, the abrupt collapse of autonomic tone leads to vasodilation, hypotension, and dependence on high-dose vasopressors to maintain perfusion pressure. Vasopressor exposure, while necessary to preserve organ viability, may impair myocardial perfusion at the microvascular level and contribute to metabolic derangements and impaired myocardial reserve. At the time of implantation, DBD hearts may appear grossly edematous due to aggressive volume resuscitation in the donor ICU, making surgical handling and anastomosis more challenging. This interstitial edema also reduces ventricular compliance, contributing to elevated filling pressures and biventricular diastolic dysfunction in the early postoperative period.

Inflammation further complicates the perioperative course in DBD transplantation. Brain death induces a systemic inflammatory response, characterized by elevated cytokines, endothelial activation, and leukocyte adhesion molecule expression. These changes not only prime the graft for enhanced alloimmune recognition but also disrupt normal endothelial function during reperfusion. At the time of graft revascularization, the inflammatory burden may contribute to transient vasoplegia, impaired coronary reflow, and the development of myocardial stunning—a reversible form of systolic dysfunction requiring pharmacologic support.

### Immunological Risk and Rejection Patterns

The immunologic consequences of heart transplantation are heavily influenced by the graft’s pre-implantation immune landscape, which may be shaped in part by the donor’s cause of death. Differences in inflammatory and endothelial activation at the time of procurement modulate antigen presentation, innate immune activation, and the susceptibility of the graft to recipient alloimmune responses [20]. These factors contribute to varying patterns of acute and chronic rejection, as well as differences in immunologic surveillance needs and responsiveness to immunosuppression [21].

### Innate and Adaptive Immune Activation

**Figure 3:**
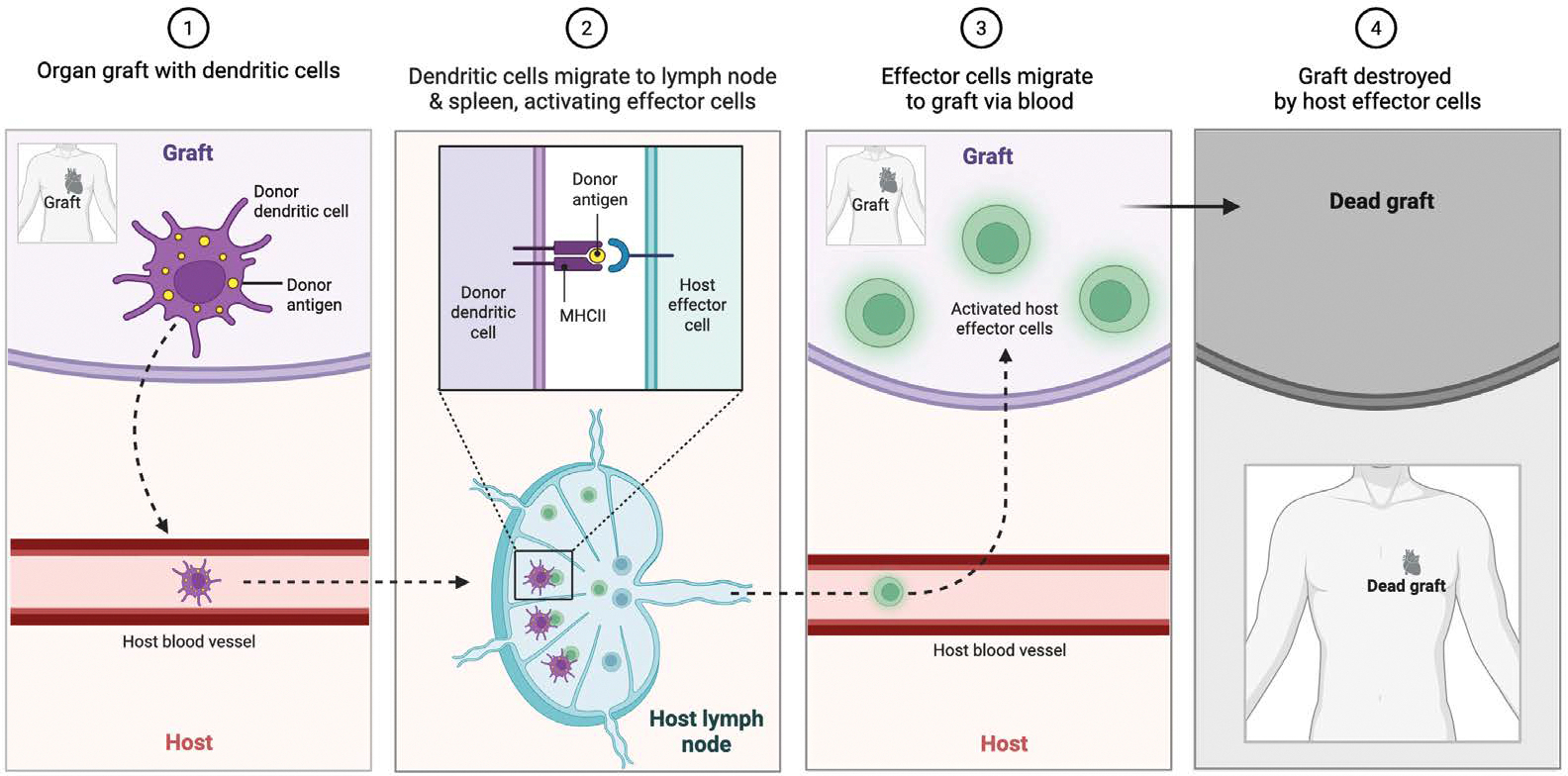
Following transplantation, donor antigen-presenting cells (APCs)—such as dendritic cells—migrate from the graft to the recipient’s lymphoid tissues. There, they directly present donor MHC-antigen complexes to host T cells, initiating a cytotoxic immune response. Activated effector cells subsequently traffic to the graft and mediate tissue destruction, leading to graft failure.

Upon transplantation, the donor heart is recognized by the recipient’s immune system through both direct and indirect antigen presentation pathways [22]. The direct pathway involves intact donor antigen-presenting cells (APCs), such as dendritic cells and macrophages, migrating to the recipient’s lymphoid tissue and stimulating T cells via donor MHC molecules. The indirect pathway, by contrast, relies on recipient APCs that process and present donor-derived peptides on self-MHC (Figure 3).

Hearts procured from inflamed donors—more commonly observed in brain death—are more likely to harbor activated graft-resident APCs, upregulated HLA class I and II molecules, and increased expression of co-stimulatory signals (CD80/CD86) [20]. This immunologically “hot” graft microenvironment facilitates early T cell priming and increases the likelihood of acute cellular rejection. Moreover, donor endothelial activation enhances complement deposition, increasing susceptibility to antibody-mediated rejection (AMR) in recipients with pre-formed donor-specific antibodies (DSAs).

In contrast, DCD grafts, although subject to ischemia-reperfusion injury, may have fewer viable donor leukocytes at the time of implantation due to the period of warm ischemia and ensuing cell death. This may theoretically reduce direct antigen presentation and early T cell priming. However, necrotic injury releases damage-associated molecular patterns (DAMPs) such as HMGB1 and mitochondrial DNA, which activate innate immune receptors including TLRs and NLRP3 inflammasomes. This sterile inflammation may still provoke alloimmunity indirectly, even in the absence of viable donor APCs [21].

### Clinical Rejection Phenotypes and Patterns

Acute cellular rejection (ACR) remains the most common form of rejection in the early post-transplant period [23]. It is driven by recipient CD4+ and CD8+ T cells targeting donor myocytes and interstitial tissue, resulting in lymphocytic infiltration, myocyte necrosis, and impaired systolic function. The clinical presentation can range from asymptomatic histologic findings to fulminant heart failure. Surveillance endomyocardial biopsies graded by ISHLT criteria (e.g., 1R to 3R) remain the standard for detection.

Studies have reported slightly higher rates of early ACR in recipients of DBD grafts, potentially due to enhanced antigenicity of inflamed tissue [21]. This may manifest as recurrent borderline rejection or 1R episodes in the first 3–6 months post-transplant, even under standard immunosuppression [24]. However, modern protocols and routine surveillance have largely equalized ACR outcomes between DBD and DCD recipients in high-volume centers.

Antibody-mediated rejection (AMR), mediated by DSAs binding to donor endothelial antigens, results in complement activation, capillary inflammation, and microvascular dysfunction [20]. The presence of C4d deposition in capillaries on biopsy and elevated donor-specific antibody titers supports the diagnosis. AMR may be hyperacute, occurring within hours in recipients with pre-formed DSAs and inadequate crossmatch, or subacute/chronic, developing insidiously over weeks.

While AMR is strongly influenced by recipient sensitization, donor-related factors may contribute. Inflammatory endothelial activation in DBD grafts may increase the density and accessibility of alloantigens such as HLA-DR or HLA-DQ on endothelial surfaces. This can potentiate DSA binding and amplify complement-mediated injury. In contrast, ischemia-reperfusion in DCD may also expose neoantigens or induce expression of non-HLA targets (e.g., angiotensin receptor type 1), triggering atypical AMR phenotypes.

### Chronic Immune-Mediated Injury

Long-term graft survival is limited not only by episodic rejection but also by insidious chronic immune activation. The leading manifestation is cardiac allograft vasculopathy (CAV), a progressive fibroproliferative disease of the coronary arteries [25]. CAV is thought to result from both immune-dependent mechanisms (e.g., low-grade T-cell and B-cell activation) and non-immune factors such as metabolic syndrome and donor age. Grafts procured under inflammatory conditions, such as DBD, may be predisposed to early endothelial dysfunction and intimal hyperplasia, creating a fertile substrate for CAV development. While long-term CAV rates in DCD recipients remain under investigation, preliminary reports suggest that with optimal preservation strategies, rates are not substantially different [26].

### Implications for Immunosuppression and Surveillance

**Figure 4:**
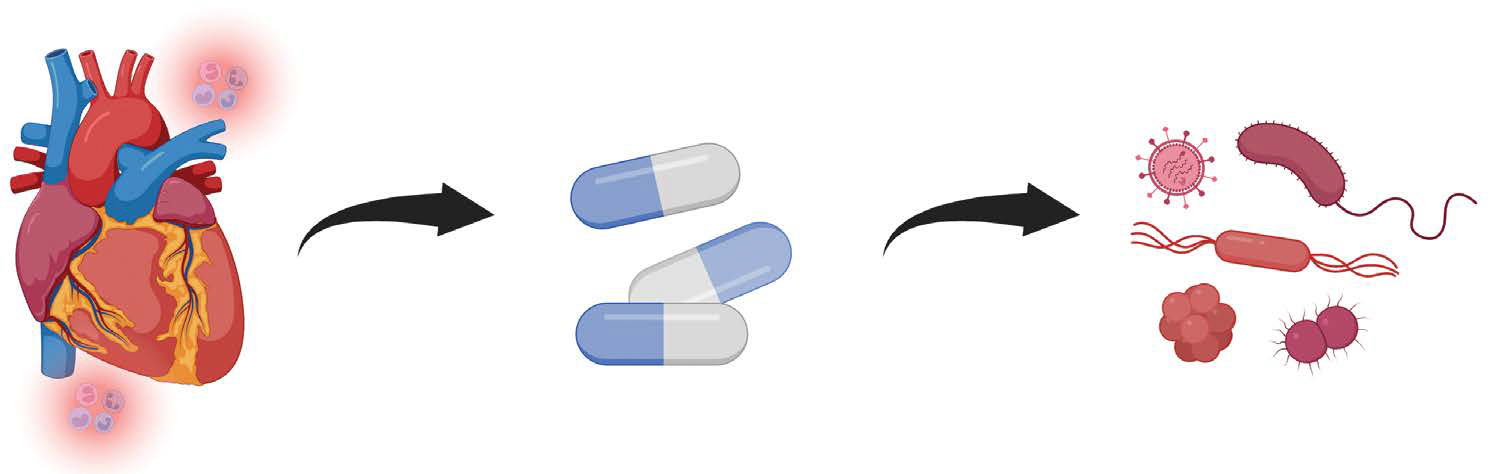
To prevent allograft rejection, heart transplant recipients require potent immunosuppressive therapy. While these medications reduce immune-mediated injury to the donor heart, they also impair host defenses, increasing susceptibility to hospital-acquired and opportunistic infections. This balance between rejection prevention and infection risk is a central challenge in post-transplant management.

Given the immunogenic profile of DBD grafts, some centers may consider more aggressive induction therapy (e.g., antithymocyte globulin or basiliximab) in recipients at higher risk for rejection. In contrast, DCD recipients may benefit from early graft function monitoring and ischemia-reperfusion mitigation rather than heightened immunosuppression. The evolving use of non-invasive rejection biomarkers, such as donor-derived cell-free DNA and gene expression profiling, may offer enhanced sensitivity to immune activation regardless of donor type and may one day permit donor-specific tailoring of rejection surveillance [27–29] (Figure 4).

Ultimately, donor death physiology interacts with recipient immune status, crossmatch results, and immunosuppressive regimen to shape the trajectory of graft adaptation or injury [30]. Appreciating the unique immunologic fingerprints left by each donor type enables more nuanced risk assessment and immunologic vigilance in the post-transplant period.

### Infection Risk and Long-Term Complications

Heart transplant recipients remain vulnerable to a spectrum of infectious and chronic complications that may impair graft longevity and patient survival [31]. While many of these risks are driven by immunosuppressive therapy, comorbidities, and recipient immune status, emerging evidence suggests that donor-related factors—including the cause of death—may influence early immune surveillance, endothelial integrity, and susceptibility to injury [32]. Understanding how these variables affect infection risk, chronic rejection, cardiac allograft vasculopathy, and post-transplant malignancy is critical to optimizing long-term outcomes [25].

### Infection Risk in the Early Post-Transplant Period

Infectious complications remain one of the leading causes of early morbidity and mortality in heart transplant recipients, especially within the first 90 days post-operatively when immunosuppression is at its highest [31]. While infections are primarily attributed to opportunistic pathogens, surgical exposure, and impaired host defenses, the condition of the donor heart at the time of procurement can play a contributory role (Figure 4).

Hearts procured from DBD donors, particularly those with prolonged ICU stays, are often colonized with hospital-acquired flora, including multidrug-resistant organisms such as Pseudomonas aeruginosa, Klebsiella pneumoniae, or MRSA [33]. Additionally, systemic inflammation in the donor may impair the function of graft-resident immune cells, such as tissue macrophages, neutrophils, and endothelial cells, which ordinarily contribute to pathogen recognition and containment [32]. This immunologic dysfunction, compounded by ischemia-reperfusion stress and early corticosteroid exposure, may facilitate bacterial translocation, viral reactivation (e.g., CMV, EBV), or donor-derived infections (Figure 4).

In DCD transplantation, the period of warm ischemia may compromise barrier integrity and cellular immune function within the graft [34]. Although DCD donors often have shorter ICU stays and reduced exposure to nosocomial pathogens, the metabolic injury associated with ischemia-reperfusion can result in capillary leak, reduced antimicrobial peptide production, and endothelial vulnerability, all of which may increase susceptibility to bloodstream infections, pneumonia, and surgical site infections. Clinical studies comparing DBD and DCD recipients have not consistently demonstrated major differences in infection rates, though microbial culture patterns and timing of onset may vary [23].

Importantly, early infections may delay weaning from mechanical ventilation, impair wound healing, and necessitate interruptions in immunosuppression, increasing the risk for both rejection and prolonged hospitalization. Individualized antimicrobial prophylaxis, careful screening of donor microbiological status, and early initiation of preemptive therapy for high-risk viruses (e.g., CMV in mismatched recipients) remain essential components of post-transplant care.

### Cardiac Allograft Vasculopathy

**Figure 5:**
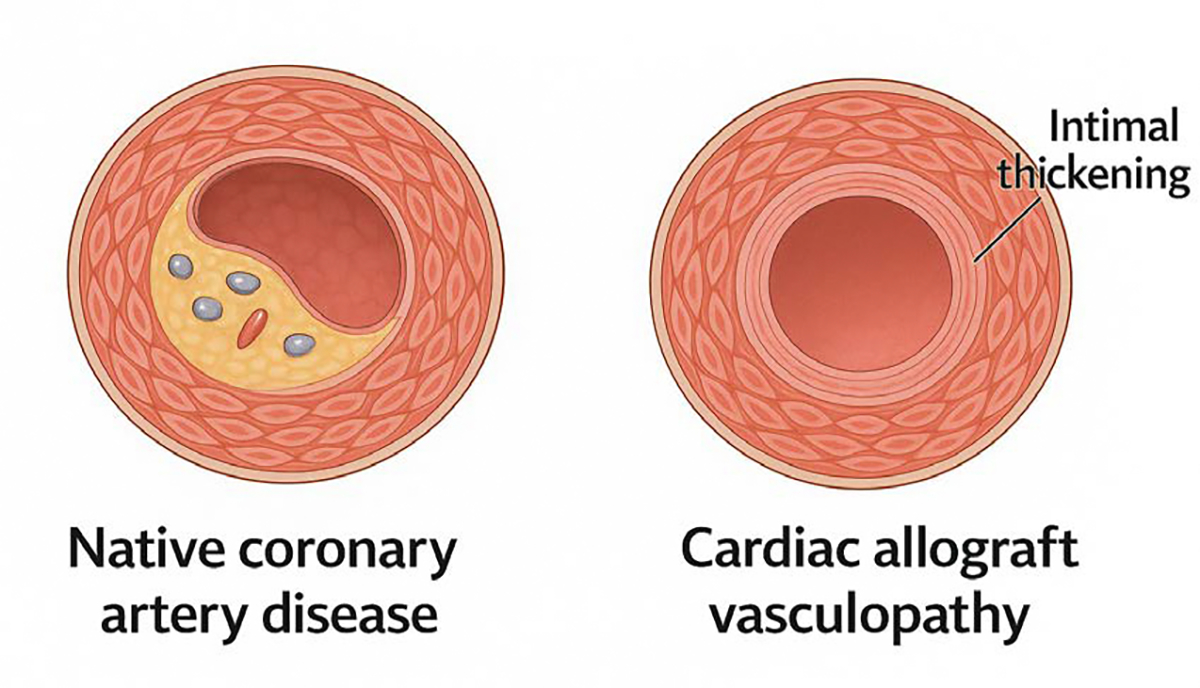
Native coronary artery disease (CAD) is characterized by focal plaque buildup with lipid accumulation and inflammatory cell infiltration. In contrast, cardiac allograft vasculopathy (CAV) involves diffuse, concentric intimal thickening throughout the coronary vasculature, driven by immune-mediated endothelial injury and chronic inflammation. Unlike CAD, CAV progresses silently and often affects distal vessels, posing a major cause of late graft failure.

Cardiac allograft vasculopathy (CAV) is the most common form of chronic allograft failure and a major contributor to late mortality in heart transplant recipients. It is characterized by diffuse concentric intimal thickening of epicardial and intramyocardial coronary arteries, driven by chronic immune activation, endothelial injury, and maladaptive remodeling. Unlike native coronary artery disease, CAV affects both large and small vessels uniformly, making it difficult to detect with conventional angiography in its early stages (Figure 5).

Although DCD grafts are also subject to injury—particularly from oxidative stress and reperfusion damage—the extent of baseline endothelial activation may be lower. Limited longitudinal studies suggest that when ischemic time is well controlled and preservation techniques such as normothermic perfusion are used, CAV incidence at 5 years is comparable between DCD and DBD recipients. However, larger and longer-term datasets are needed to clarify whether subtle differences in vascular injury patterns lead to divergent outcomes beyond the first post-transplant decade.

Risk factors that compound donor-related risk include older donor age, pre-existing coronary disease, recipient hyperlipidemia, diabetes, and episodes of rejection, particularly AMR [35]. Serial surveillance with intravascular ultrasound (IVUS), optical coherence tomography (OCT), or advanced perfusion imaging can identify early CAV before clinical symptoms appear, allowing for intensified lipid-lowering, immunosuppressive adjustment, or targeted anti-inflammatory therapies [36].

### Chronic Rejection and Interstitial Fibrosis

Beyond vascular pathology, chronic rejection can also manifest as progressive interstitial fibrosis and myocardial stiffening, ultimately leading to diastolic dysfunction and graft failure. Low-level, persistent immune activation—often subclinical—driven by minor histocompatibility mismatches or unresolved donor inflammation may contribute to fibrogenic signaling pathways, particularly in the myocardium and perivascular regions [37]. Transforming growth factor-beta (TGF-β), matrix metalloproteinases, and fibroblast proliferation are central mediators of this process [38].

This phenomenon may be more pronounced in DBD grafts, especially those with recurrent subclinical cellular rejection, repeated allograft injury from hypotension, or inflammatory priming [39]. The role of DCD grafts in chronic myocardial remodeling is less clear but may involve distinct pathways related to hypoxic injury, iron deposition, or impaired lymphatic drainage following reperfusion [40].

### Malignancy Risk and Immune Surveillance

Long-term immunosuppression in heart transplant recipients increases the risk of de novo malignancies, particularly skin cancers, lymphomas (including post-transplant lymphoproliferative disorder [PTLD]), and solid tumors [41–42]. While the development of malignancy is primarily related to cumulative immunosuppressive exposure and oncogenic viral reactivation (e.g., EBV), it is plausible that donor physiology may influence early immune surveillance capacity [43].

In DBD recipients, where graft-resident antigen-presenting cells may be more activated, early immune exhaustion or compensatory suppression might blunt tumor immune surveillance, though this remains speculative. DCD grafts, having fewer viable immune cells at the time of implantation, may result in different kinetics of immune reconstitution and T-cell repopulation [40]. However, current evidence does not support a strong or consistent link between donor death mechanism and long-term cancer risk [41].

### Orthotopic vs. Heterotopic Heart Transplantation

Most heart transplants performed today utilize the orthotopic technique, in which the recipient’s diseased heart is removed and replaced entirely by the donor organ [44]. However, heterotopic heart transplantation (HHT)—in which the donor heart is implanted alongside the native heart in a parallel or assist configuration—remains an important, though rarely used, alternative in select patients [45]. Each approach presents unique surgical, physiological, and long-term implications, particularly when considering patient-specific factors such as pulmonary vascular resistance, myocardial recovery potential, and anatomical constraints [40].

### Orthotopic Heart Transplantation

In orthotopic heart transplantation (OHT), the native heart is excised, and the donor heart is implanted in its anatomical position. The surgical anastomoses typically include the left atrial cuff, pulmonary artery, aorta, and sometimes the superior and inferior vena cava, depending on the technique (bicaval vs. biatrial) [46]. The procedure restores normal cardiac anatomy and allows for direct functional replacement.

This technique is appropriate for many transplant recipients and provides optimal alignment of ventricular and valvular structures [47]. Surveillance is also simplified: endomyocardial biopsies, echocardiography, and coronary angiography can be performed without anatomical interference from the native heart. Long-term outcomes are well-established, and improvements in immunosuppression and perioperative management have made OHT the standard of care [48].

However, certain physiological and anatomic conditions may render OHT suboptimal. For example, patients with severe fixed pulmonary hypertension may impose intolerable afterload on the donor right ventricle, predisposing to early right heart failure [49]. Similarly, patients with complex congenital anomalies or chest cavity limitations may not tolerate removal and replacement of the native heart. In these contexts, heterotopic transplantation offers a valuable alternative [50].

### Heterotopic Heart Transplantation

**Figure 6:**
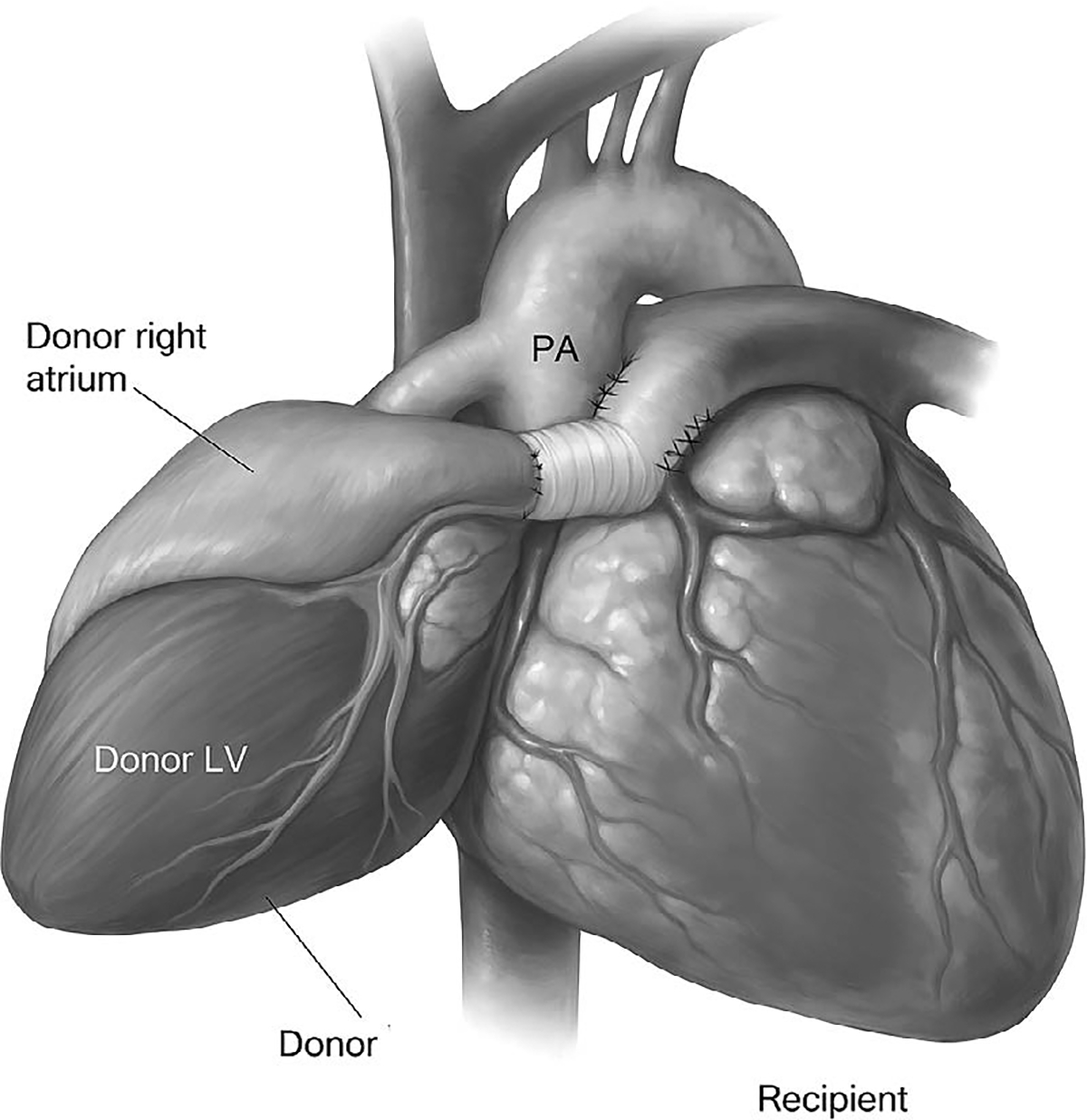
In heterotopic heart transplantation, the donor heart is implanted alongside the native heart, allowing both to contribute to systemic circulation. Vascular anastomoses connect the donor pulmonary artery and atria to the recipient’s corresponding structures. This configuration can provide mechanical support while preserving native cardiac function and is typically reserved for select patients with elevated pulmonary vascular resistance or partial myocardial recovery potential.

Heterotopic heart transplantation (HHT) involves implanting the donor heart into the right side of the thorax without removing the native heart. Vascular anastomoses are established such that the donor heart either assists or shares circulation with the native heart, depending on the specific configuration [51]. The procedure was first developed as a means of providing additional circulatory support in patients with high pulmonary vascular resistance or significant donor-recipient size mismatch (Figure 6).

In the parallel (biventricular) configuration, systemic and pulmonary circulations are shared between the native and donor hearts. The donor left atrium is anastomosed to the native left atrium, the donor pulmonary artery to the native pulmonary artery, and the aortas are joined side-to-side. Both ventricles contribute to cardiac output. This configuration can offload the native heart while preserving some of its contractile contribution, particularly in cases where native function may be partially recoverable (Figure 6).

Alternatively, in an assist configuration, the donor heart primarily supports left ventricular output, functioning analogously to a biological ventricular assist device. This setup may be chosen when native right heart function is preserved but the left ventricle has failed, such as in selected cases of peripartum cardiomyopathy or myocarditis.

A notable advantage of HHT is the potential for native heart recovery. In cases of reversible myocardial dysfunction, the preserved native heart may gradually regain contractility over time. If recovery is sufficient, the donor heart can be electively explanted, effectively making the transplant a temporary bridge to recovery rather than permanent replacement [52]. While such cases are rare, they represent a meaningful clinical success, particularly in young patients with acute, reversible cardiomyopathy.

However, heterotopic transplantation is associated with unique challenges. The dual-heart anatomy complicates imaging interpretation and surveillance. Endomyocardial biopsies require precise targeting of the donor heart, and echocardiography must distinguish between native and donor contributions [53]. Furthermore, the risk of thrombus formation within the underfilled native ventricles is high, necessitating long-term anticoagulation [54]. Arrhythmias in the native heart may persist or even worsen due to altered electrical conduction and chamber stretch. There is also an increased risk of infection, bleeding, and mediastinal compression due to the enlarged cardiac silhouette.

Despite these limitations, HHT remains a lifesaving option in selected patients. In particular, recipients with irreversible pulmonary hypertension or mismatched body size may derive significant benefit from HHT when orthotopic transplantation would result in early graft failure [53]. The procedure also offers flexibility in centers without access to mechanical circulatory support devices or in settings where such devices are contraindicated.

Long-term outcomes following HHT are variable and heavily dependent on recipient selection and postoperative management. Survival may be limited by thromboembolic events, arrhythmias, or progressive failure of the donor heart. Nonetheless, careful monitoring and individualized management can lead to excellent functional status in a subset of patients.

### Clinical Considerations

The choice between orthotopic and heterotopic transplantation is ultimately determined by a combination of recipient-specific hemodynamic parameters, anatomical feasibility, and donor heart characteristics. For patients with elevated pulmonary vascular resistance, heterotopic transplantation may offer a more stable postoperative course, preventing right ventricular overload [55]. In contrast, patients with low panel reactive antibody levels, good surgical anatomy, and normal pulmonary pressures are ideal candidates for standard orthotopic implantation [56].

As modern mechanical circulatory support systems become more widely available and durable, the role of HHT has diminished. However, its utility in select scenarios—especially in patients with native heart recovery potential—warrants continued familiarity with the technique, particularly in complex or high-risk transplant cases [55].

### Recipient Demographics and Modifying Factors

The success of heart transplantation depends not only on donor quality and surgical technique but also on the baseline characteristics, physiological reserve, and risk profile of the recipient. While considerable attention is often placed on donor selection—especially the physiological consequences of brain death or circulatory arrest—recipient-specific factors interact closely with donor characteristics, influencing early graft adaptation, long-term survival, and the likelihood of complications [57]. A comprehensive understanding of how recipient demographics, comorbidities, immunologic status, and transplant candidacy criteria affect outcomes is essential to optimizing the transplant process [58].

### Age, Sex, and Comorbid Burden

Recipient age is an important modifier of transplant outcomes. Older recipients tend to have reduced myocardial reserve, slower recovery from surgery, and increased susceptibility to infection and malignancy, all of which contribute to higher early and late mortality. Advanced age is also associated with diminished physiologic adaptability to marginal donor hearts, including those subjected to ischemia or inflammation. Conversely, younger recipients generally tolerate higher immunologic activation and are better candidates for higher-risk grafts, such as DCD hearts with prolonged ischemic times [59].

Sex differences can also influence transplant outcomes, particularly in donor-recipient sex mismatch. For example, female donor to male recipient transplants have been associated with higher rejection rates and decreased survival, potentially due to size mismatch, immunologic differences in antigen expression, or increased endothelial activation [60]. Hormonal and immunological factors may further modulate graft tolerance, though the mechanisms remain incompletely understood.

Comorbid conditions such as diabetes mellitus, chronic kidney disease, hepatic dysfunction, and pulmonary disease significantly affect perioperative risk and long-term morbidity [61]. These comorbidities may reduce the physiologic buffer against PGD, limit the safety margin of immunosuppressive regimens, and contribute to cumulative end-organ injury. For instance, recipients with renal insufficiency may not tolerate calcineurin inhibitors well and are at increased risk for acute tubular injury in the perioperative period.

### Pulmonary Hemodynamics and Right Ventricular Load

Elevated pulmonary vascular resistance (PVR) is one of the most important hemodynamic predictors of early graft failure, especially in orthotopic transplantation [62]. If the donor right ventricle is exposed to a high afterload in a recipient with uncorrected pulmonary hypertension, acute right ventricular failure may ensue, often requiring mechanical support [63]. Although temporary pulmonary vasodilators (e.g., inhaled nitric oxide, prostacyclin) can reduce PVR, values persistently >4–5 Wood units despite vasodilator challenge are considered a relative contraindication to OHT [64]. In such cases, heterotopic transplantation or mechanical support may be more appropriate.

Recipients with borderline or reversible elevations in pulmonary pressures may still be considered for OHT, but donor selection becomes critical [64]. Hearts from DBD donors with preexisting right ventricular strain or DCD donors with delayed right heart recovery may not tolerate increased afterload, especially in older or marginal grafts.

### Absolute and Relative Contraindications

**Figure 7:**
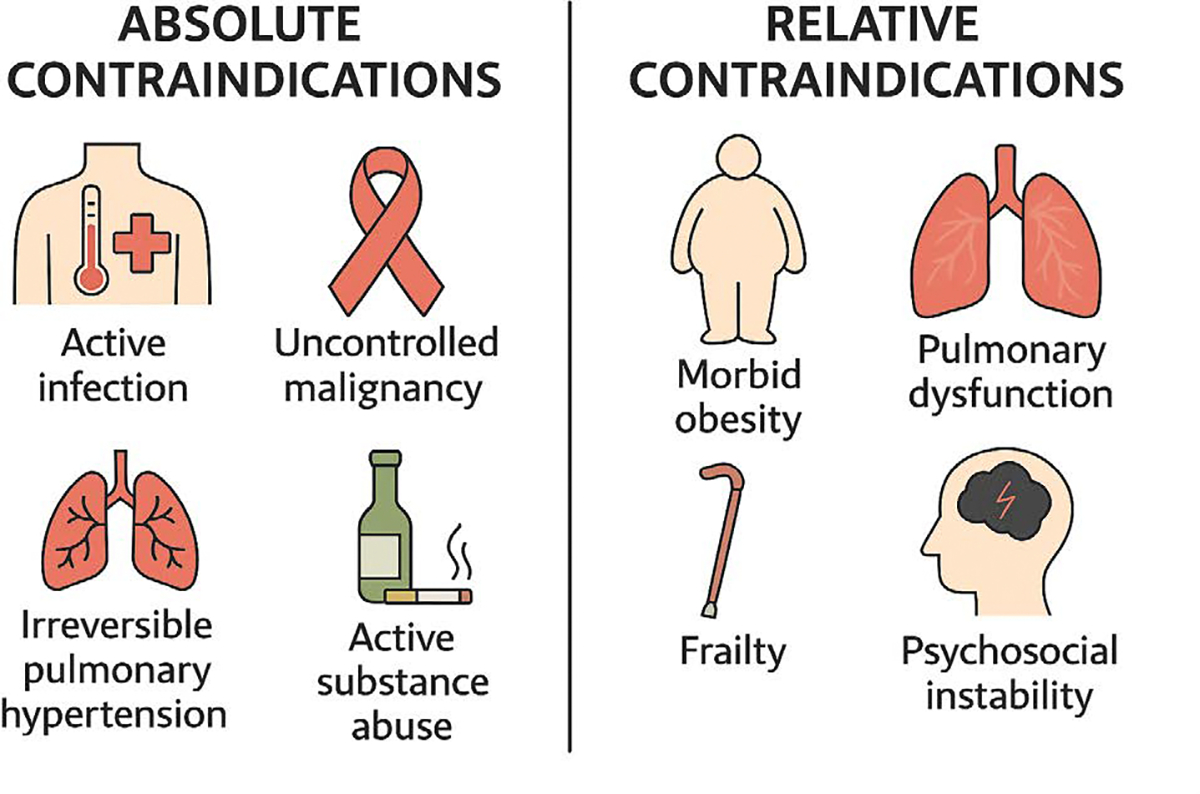
Heart transplant candidacy is determined by a careful assessment of absolute and relative contraindications. Absolute contraindications—such as active infection, uncontrolled malignancy, irreversible pulmonary hypertension, and active substance abuse—pose significant risks for perioperative mortality or graft failure. Relative contraindications, including morbid obesity, pulmonary dysfunction, frailty, and psychosocial instability, may compromise long-term outcomes but require individualized risk–benefit analysis.

Transplant candidacy is further defined by well-established absolute and relative contraindications. Absolute contraindications include active systemic infection, uncontrolled malignancy, irreversible pulmonary hypertension, and active substance abuse [65]. These conditions substantially increase the risk of perioperative death, graft failure, or nonadherence (Figure 7).

Relative contraindications are more nuanced and include conditions such as morbid obesity (BMI >35–40), frailty, moderate pulmonary dysfunction, and psychosocial instability [64]. While not exclusionary on their own, these factors may reduce the probability of success and must be weighed carefully against donor risk factors. For example, an obese or frail recipient may not tolerate prolonged cardiopulmonary bypass or may face increased complications from marginal grafts with borderline function [66]. Conversely, low-risk recipients with reversible comorbidities are more appropriate candidates for DCD grafts or donor hearts with longer ischemic times.

### Immunologic Risk and Sensitization

The recipient’s immunologic profile plays a decisive role in transplant success. Recipients with high panel reactive antibody (PRA) levels, prior blood transfusions, pregnancy, or previous transplants are considered sensitized and are at increased risk for hyperacute or antibody-mediated rejection [67]. These patients require more stringent donor selection with negative crossmatching and may benefit from desensitization therapies prior to transplant [68].

In the context of donor death physiology, a sensitized recipient may be less tolerant of DBD-associated endothelial activation, which enhances antigen presentation and complement activation. Similarly, ischemia-reperfusion injury in DCD grafts may expose neoantigens or alter immune recognition [68]. Sensitized recipients may therefore require better-matched grafts and closer post-transplant surveillance.

### ABO Blood Type and Waitlist Dynamics

ABO compatibility remains a non-negotiable requirement in adult heart transplantation, and blood type O recipients face a disproportionate burden [69]. Since type O individuals can only receive organs from type O donors—while type O donors are universal for all recipients—O recipients often experience longer waitlist times and higher pre-transplant mortality [70]. These dynamics can lead to increased risk of clinical deterioration, which, in turn, limits tolerance for marginal donor hearts [71]. When DCD utilization is considered for expanding access, waitlist prioritization for blood type O recipients may offer the greatest benefit, provided that ischemic time is minimized and post-transplant support is readily available [69].

### Interaction With Donor Characteristics

The interplay between recipient and donor factors cannot be overstated. A high-risk recipient—such as one with obesity, frailty, or elevated PVR—should ideally receive a low-risk donor organ with short ischemic time, minimal inotropic support, and no evidence of myocardial dysfunction [72]. Conversely, young, healthy recipients may tolerate extended criteria grafts, including DCD hearts or those from older DBD donors with modest instability. This individualized matching strategy reduces the risk of PGD, rejection, and early mortality.

Emerging approaches such as composite risk scores, frailty indices, and biomarker-based stratification tools are increasingly used to quantify recipient vulnerability and guide organ acceptance decisions [73]. Future directions may include integrating recipient and donor physiology into machine learning algorithms that dynamically assess transplant suitability based on real-time data.

### Recipient Evaluation and Risk Stratification

The decision to proceed with heart transplantation hinges on a rigorous and multidisciplinary evaluation of the recipient. This process not only determines candidacy but also guides donor selection, perioperative planning, and long-term management. As donor utilization expands to include hearts from DCD donors and marginal DBD donors, precise recipient risk stratification becomes even more essential [74]. The evaluation synthesizes hemodynamic, metabolic, immunologic, and psychosocial parameters to predict how a given recipient will tolerate both the surgery and the donor heart’s physiological condition.

### Hemodynamic and Functional Assessment

A foundational component of recipient evaluation is the measurement of cardiopulmonary hemodynamics via right heart catheterization. Key parameters include pulmonary artery pressure, pulmonary capillary wedge pressure, cardiac output, and most critically, pulmonary vascular resistance (PVR) [75]. Persistent elevation in PVR, particularly values above 4–5 Wood units that fail to respond to vasodilator challenge, indicates a high-risk for post-transplant right ventricular failure, particularly if a marginal donor heart or DCD graft is used. In such cases, the transplant team must consider either intensified perioperative support or alternative strategies such as heterotopic transplantation.

In parallel, functional capacity is assessed through objective measures such as cardiopulmonary exercise testing (peak VO_2_ <14 mL/kg/min) or a six-minute walk test. These tools help quantify the severity of heart failure and the degree of deconditioning, which may influence graft recovery and rehabilitation potential [76]. Frailty assessments—encompassing grip strength, gait speed, and nutritional status—are increasingly recognized as independent predictors of post-transplant morbidity and mortality.

### End-Organ Function and Systemic Reserve

Pre-transplant evaluation must also assess the viability of non-cardiac organs, particularly the kidneys, liver, lungs, and brain, as their dysfunction may complicate both the procedure and immunosuppressive therapy. For example, chronic kidney disease (eGFR <40 mL/min/1.73 m^2^) may necessitate simultaneous heart-kidney transplantation, while liver dysfunction with evidence of portal hypertension or synthetic failure could contraindicate isolated heart transplantation altogether [77].

Pulmonary function testing is crucial to identify obstructive or restrictive lung disease that might impair postoperative ventilation [78]. Similarly, cerebrovascular imaging and neurocognitive assessment are indicated in older patients or those with prior strokes to evaluate procedural risk and the capacity for informed consent and adherence.

### Immunologic Evaluation

The immunologic profile of the recipient determines susceptibility to antibody-mediated rejection (AMR) and guides donor selection. This includes testing for panel reactive antibodies (PRA), donor-specific antibodies (DSAs), and HLA sensitization [79]. Sensitized recipients are at elevated risk for hyperacute or early AMR and require virtual or prospective crossmatching to confirm compatibility [80]. Preformed DSAs may also influence immunosuppression intensity or eligibility for desensitization protocols prior to transplant [81].

Moreover, recipients with autoimmune diseases, prior transplants, or high PRA values may not be optimal candidates for DCD grafts, in which ischemic injury can trigger heightened alloimmune responses [82]. In such patients, the transplant team may prioritize DBD donors with low ischemic times and stable hemodynamics to reduce immunologic priming.

### Psychosocial Evaluation and Adherence Potential

A comprehensive psychosocial evaluation is a mandatory component of transplant candidacy. This includes an assessment of mental health, substance use history, social support networks, and health literacy. Post-transplant success depends on strict medication adherence, attendance at frequent follow-up appointments, and early symptom recognition—all of which require reliable patient engagement [83]. Active substance use, untreated psychiatric illness, or lack of caregiver support may constitute absolute or relative contraindications.

Financial and logistical access to care must also be confirmed, particularly for access to immunosuppressive medications, travel to transplant centers, and routine laboratory monitoring [84]. Multidisciplinary teams—including social workers, psychiatrists, and transplant coordinators—play an essential role in mitigating modifiable psychosocial risks [85].

### Risk Stratification Tools

**Figure 8:**
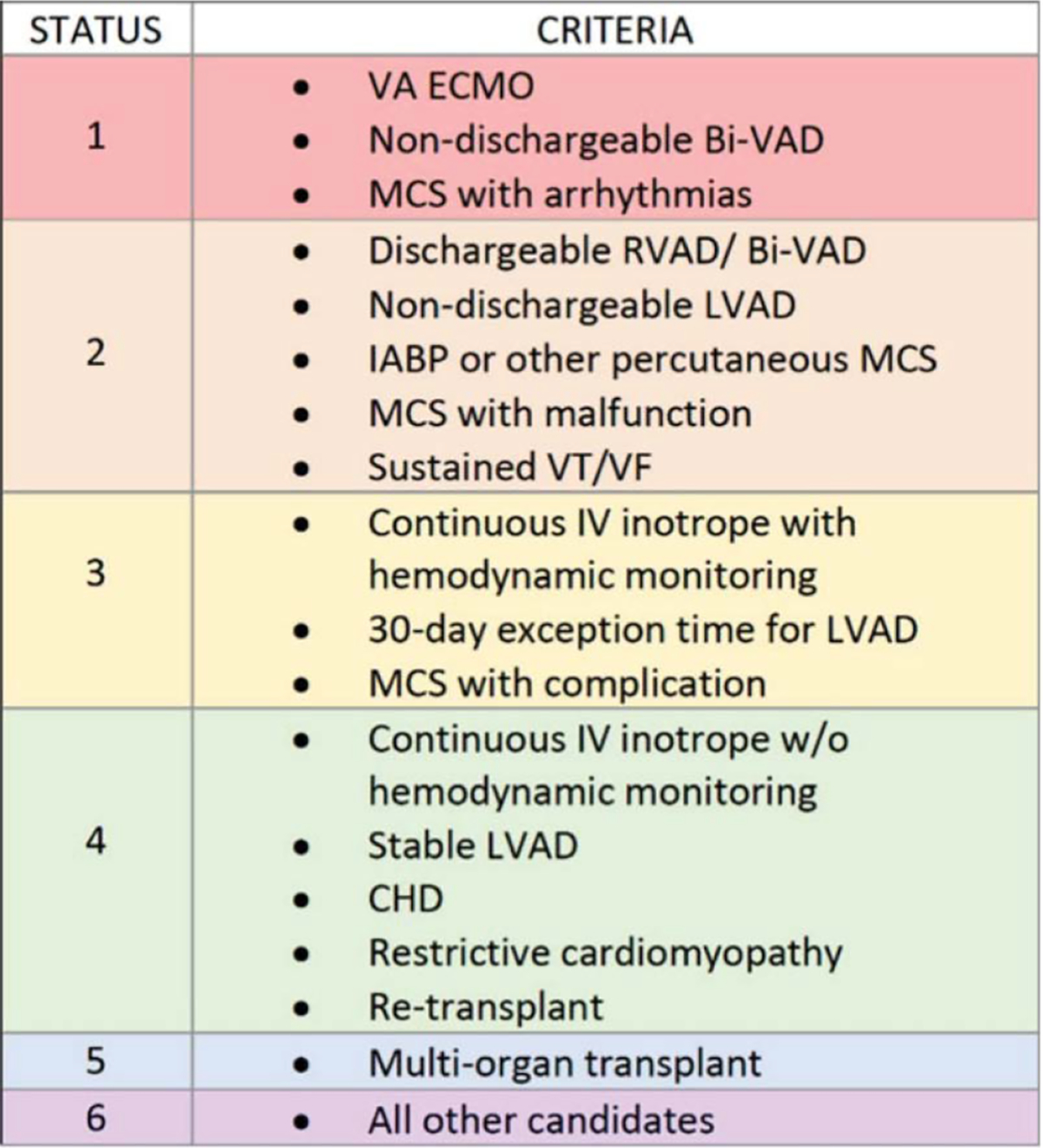
The United Network for Organ Sharing (UNOS) categorizes adult heart transplant candidates into six statuses based on clinical urgency. Status 1 represents the most critical patients—those requiring VA ECMO or non-dischargeable biventricular mechanical support—while Status 6 includes stable candidates without advanced support needs. This stratification guides equitable organ allocation by prioritizing those with the highest medical urgency and likelihood of benefit.

Several scoring systems have been developed to stratify recipient risk and inform organ allocation decisions: (i) The IMPACT score (Index for Mortality Prediction After Cardiac Transplantation) uses recipient age, mechanical support status, ventilator dependence, bilirubin, and other variables to predict one-year mortality. A higher IMPACT score may discourage the use of marginal donors or prolonged ischemic time [86]. (ii) INTERMACS profiles, originally developed for advanced heart failure and mechanical circulatory support, provide a seven-level functional scale that correlates with transplant urgency [87]. INTERMACS levels 1–2 reflect critical cardiogenic shock and often require immediate transplantation or bridging with ECMO or LVAD. (iii) The United Network of Organ Sharing (UNOS) heart allocation system further prioritizes recipients based on severity of illness, mechanical support requirements, and other dynamic clinical variables (Figure 8). Status 1 patients typically require the most urgent access to donor organs and may be prioritized for standard DBD donors over higher-risk DCD grafts [88].

Emerging tools include frailty scores, biomarker-based prediction models, and machine learning algorithms that integrate multidimensional clinical data to more accurately forecast outcomes [86].

### Integration With Donor Characteristics

Risk stratification is most impactful when coupled with an understanding of donor physiology. For example, a low-risk recipient with robust end-organ function and minimal sensitization may tolerate a donor heart exposed to moderate warm ischemia or vasopressor support [89]. In contrast, high-risk or borderline candidates should be matched to ideal donor hearts with minimal ischemic injury, short transport times, and stable hemodynamics [90].

This dynamic matching process requires real-time communication between procurement teams, surgical teams, and transplant physicians [91]. The increasing use of donor risk indices, such as the UNOS-derived DRI or OHT-specific predictive tools, further supports rational allocation and risk mitigation [92].

### Emerging Biomarkers and Technologies

The integration of advanced biomarkers, imaging modalities, and organ preservation systems is transforming the field of heart transplantation. These technologies enable earlier detection of graft injury, improved immunologic surveillance, and more precise decision-making around donor selection—particularly when evaluating hearts from donors after brain death (DBD) or circulatory death (DCD) [93–94]. As transplantation increasingly incorporates extended criteria donors and marginal grafts, these innovations are becoming essential to mitigate risk and personalize care.

### Donor-Derived Cell-Free DNA and Gene Expression Profiling

**Figure 9:**
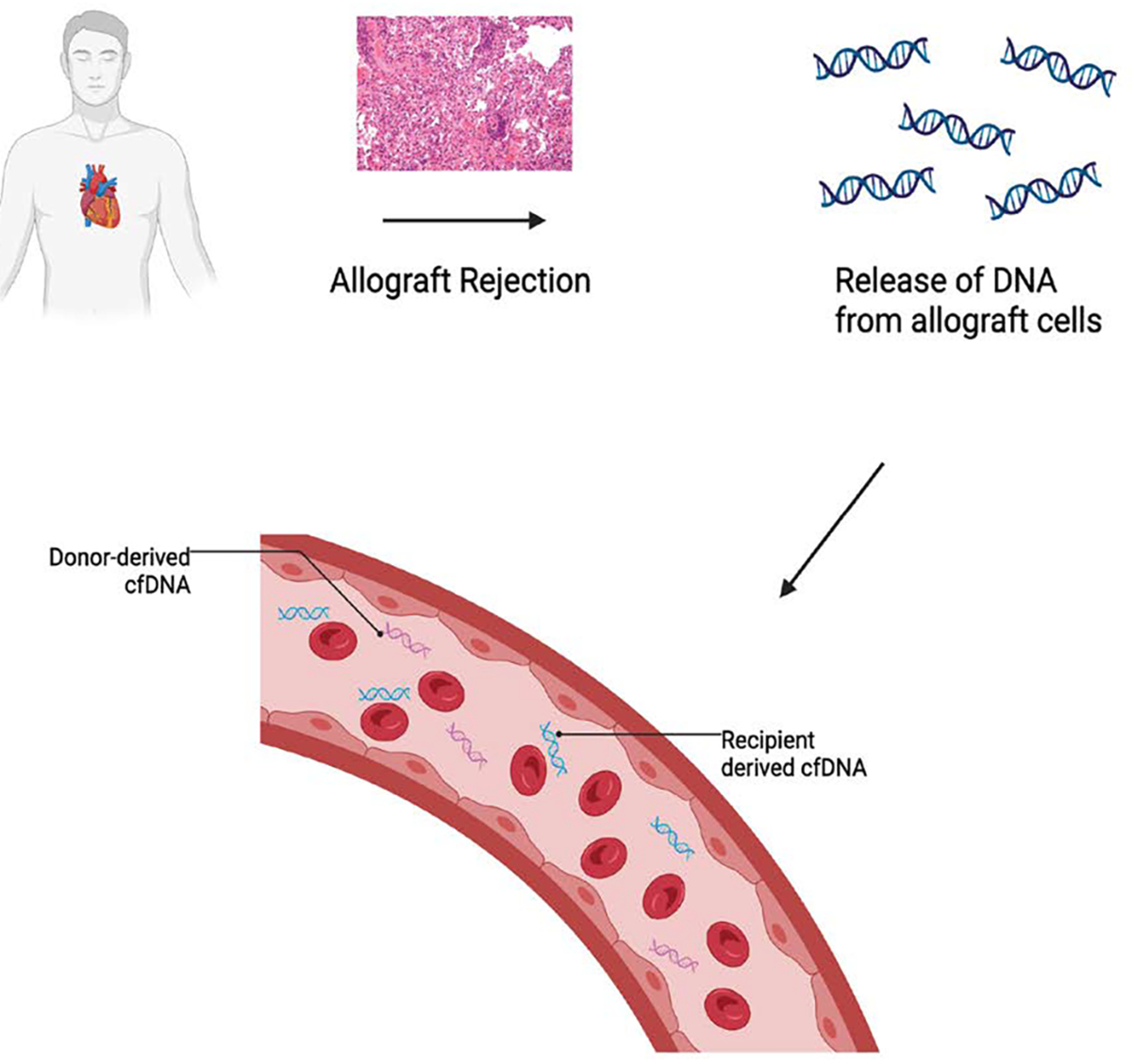
During allograft rejection, injured donor cells release fragmented DNA into the recipient’s circulation. This donor-derived cell-free DNA (dd-cfDNA) can be distinguished from recipient DNA and quantified in blood. Elevated levels of dd-cfDNA serve as a noninvasive biomarker for acute rejection, offering a promising tool for early detection and graft monitoring in heart transplant recipients.

Donor-derived cell-free DNA (dd-cfDNA) has emerged as a highly sensitive and specific biomarker for detecting allograft injury. Following transplantation, injured or apoptotic donor cells release fragments of DNA into the recipient’s circulation. Measurement of dd-cfDNA allows for real-time assessment of cellular injury, with levels rising during episodes of acute rejection, early graft dysfunction, or ischemia-reperfusion injury [95]. In the setting of DCD transplantation, where warm ischemia and reperfusion stress are common, elevated dd-cfDNA may serve as an early warning signal of subclinical damage even in the absence of histological rejection [96]. Conversely, low and stable dd-cfDNA concentrations in clinically well patients support safe minimization of immunosuppression and reduction in the frequency of surveillance biopsies [93] (Figure 9).

Gene expression profiling provides another non-invasive tool for monitoring immune activation. The AlloMap test, for instance, evaluates the expression levels of a panel of immune response genes in peripheral blood to estimate the risk of acute cellular rejection [97–98]. This assay, while primarily used for routine surveillance beyond the early post-transplant period, may be useful in clarifying ambiguous graft dysfunction—such as distinguishing rejection from ischemic injury in marginal or DCD grafts. When interpreted alongside dd-cfDNA levels, gene expression profiles add an additional layer of precision to post-transplant risk assessment (Figure 9).

### Advanced Cardiac Imaging

In addition to molecular diagnostics, advanced imaging modalities are increasingly used to monitor graft health and detect early signs of dysfunction. Cardiac magnetic resonance imaging (CMR) offers high-resolution assessment of myocardial structure, edema, fibrosis, and inflammation. In recipients of DCD hearts, CMR can identify diffuse myocardial edema or regional wall motion abnormalities that may not be apparent on echocardiography. Techniques such as T1 and T2 mapping allow for non-invasive quantification of tissue injury and can help differentiate between immune-mediated rejection and reperfusion-related damage [99–100].

Strain imaging, particularly global longitudinal strain (GLS) measured via speckle-tracking echocardiography, has also proven valuable in detecting subtle systolic impairment before changes in ejection fraction occur. Although originally validated in non-transplant populations, GLS has shown high sensitivity for subclinical myocardial dysfunction and may be extrapolated to the unique context of heart transplantation, particularly for evaluating DCD grafts in the early post-transplant period or in cases where standard echocardiographic parameters appear deceptively normal [101].

### Ex Vivo Perfusion and Functional Assessment

Normothermic ex vivo heart perfusion systems represent a major advancement in donor heart preservation. Devices such as the TransMedics Organ Care System maintain the donor heart in a warm, oxygenated, beating state during transport, simulating near-physiologic conditions [102]. This approach not only prolongs preservation time but also enables continuous monitoring of cardiac function, including contractility, coronary flow, rhythm stability, and metabolic activity [103]. These features are especially critical in DCD hearts, where in situ evaluation prior to procurement is not possible [104]. Through real-time assessment of lactate clearance and hemodynamic parameters, clinicians can more confidently determine graft suitability and anticipate perioperative support needs.

Ex vivo perfusion has also demonstrated potential to reverse ischemic injury acquired during procurement, acting as a platform for myocardial recovery and reconditioning. In marginal DBD hearts with myocardial stunning or metabolic acidosis, perfusion systems can restore function, making previously unusable grafts viable for transplantation [105].

### Artificial Intelligence and Predictive Modeling

With the proliferation of clinical, biomarker, and imaging data, machine learning and artificial intelligence are being applied to develop predictive models of transplant outcomes. These algorithms can integrate complex variables, including donor death mechanisms, ischemic times, recipient comorbidities, immunologic profiles, and perfusion metrics, to forecast risks such as primary graft dysfunction, acute rejection, or graft loss [106]. Early studies suggest that AI-driven tools outperform traditional scoring systems in stratifying recipients and optimizing donor-recipient matching [107].

In the future, these platforms may provide real-time decision support during organ allocation, helping clinicians determine the optimal match between donor physiology and recipient risk. They may also guide the timing and intensity of immunosuppression, identify patients who would benefit from early surveillance, and predict when a graft is approaching functional decline [108].

### Future Directions

The future of heart transplantation lies at the intersection of donor optimization, recipient personalization, and technological innovation [109]. As clinical teams face increasing complexity in donor-recipient matching and expand the use of non-traditional donor hearts, research and policy must evolve to support better outcomes and broader access [110]. The following subthemes highlight key areas where future developments are likely to reshape clinical practice.

### Optimizing Donor Management

Improving the physiological stability of donors, particularly those who are brain-dead, remains a major focus. Brain death induces a series of maladaptive responses, including a catecholamine surge, hormonal collapse, and systemic inflammation, which may predispose the donor heart to ischemic injury and immune priming [111]. Current donor management strategies—such as hormone resuscitation with vasopressin, corticosteroids, and thyroid hormone—are inconsistently applied and based on limited prospective data [112]. Future efforts must prioritize the development of standardized, evidence-based donor management protocols. These should aim to maintain optimal preload, afterload, and coronary perfusion while minimizing inflammatory injury, thereby improving graft function and expanding the usable donor pool.

### Expanding and Refining DCD Utilization

Donation after circulatory death has already demonstrated promise in expanding donor availability without compromising graft survival, especially when combined with normothermic regional perfusion or ex vivo perfusion [113]. However, widespread adoption is limited by concerns about warm ischemia, delayed graft function, and the lack of standardized assessment criteria [24]. Future research should establish precise thresholds for functional warm ischemic time and develop validated measures for intraoperative graft viability. Additional work is needed to identify which recipients—based on comorbidity burden, right heart function, or immunologic status—are most appropriate for DCD hearts. Broadening the clinical indications for DCD transplantation, along with improved procurement logistics, will be essential for scaling this approach safely [114].

### Tailoring Immunosuppression to Donor and Recipient Biology

Traditional immunosuppression protocols are largely standardized despite wide variation in both donor heart inflammation and recipient immune reactivity [115]. Future strategies will need to move toward risk-adapted approaches, in which immunosuppressive regimens are personalized based on donor injury profile and recipient immunologic risk. For example, hearts from DBD donors with significant hemodynamic instability may require intensified induction therapy or prolonged rejection surveillance [116]. Recipients with high levels of donor-specific antibodies may benefit from pre-transplant desensitization, plasma exchange, or targeted antibody therapy [117]. Future trials should stratify patients by donor physiology—especially DBD vs DCD status—and assess outcomes under differentiated immunosuppressive regimens [118].

### Integrating Non-Invasive Monitoring and Predictive Analytics

Emerging non-invasive biomarkers such as dd-cfDNA and gene expression profiling are likely to become cornerstones of routine surveillance [93]. Their ability to detect subclinical graft injury and immune activation without the risks of biopsy will fundamentally change how clinicians monitor for rejection. In parallel, the application of machine learning and predictive modeling can enhance clinical decision-making by integrating a multitude of variables—ranging from ischemic time and donor cause of death to biomarker trends and recipient frailty scores [119]. Future systems may deliver real-time risk predictions at the point of care, guiding decisions about organ acceptance, surveillance intensity, or even early intervention for anticipated complications.

### Promoting Equity and Broader Access

Despite improvements in transplant science, disparities persist in waitlist times, access to advanced therapies, and long-term outcomes. Blood type O recipients, for example, face disproportionate delays and may benefit from prioritization strategies in the allocation of DCD organs or use of universal donor technologies [69]. Expanding access to care through telemedicine platforms, regional biopsy and laboratory networks, and home-based monitoring will be critical to ensure that advances in technology benefit a diverse and geographically distributed patient population [120]. These structural innovations must be matched with culturally competent care models and systems that address social determinants of health in transplant outcomes.

### Looking Ahead: Regenerative and Adjunctive Strategies

Beyond improvements in donor and recipient matching, the long-term future of transplantation may involve regenerative approaches that reduce reliance on scarce donor organs altogether. Advances in bioengineered tissues, cardiac scaffolds, and immune-modified allografts are underway, although still largely experimental. Adjunctive therapies, such as mesenchymal stem cell infusion or gene editing of donor organs to reduce immunogenicity, are also under investigation [121]. While these approaches may take years to reach clinical utility, their development signals a broader shift toward reshaping the biological limitations of transplantation.

## Conclusions

Donor cause of death is a critical, though often underappreciated, determinant of heart transplant outcomes [122]. The contrasting pathophysiological environments associated with brain death and circulatory death impart distinct patterns of myocardial injury, inflammatory priming, and ischemic stress, each of which carries unique implications for graft function, immune response, and long-term survival. As transplantation practices evolve to include broader donor criteria—particularly the increasing use of DCD grafts—careful consideration of donor physiology has become essential for optimal donor-recipient matching and perioperative planning.

Ultimately, the field is moving toward a precision-guided model of transplantation—one that integrates donor physiology, recipient characteristics, and evolving clinical technologies to inform every stage of care [123]. Continued progress will depend on robust clinical trials, widespread adoption of best practices in donor management, and the equitable implementation of emerging tools across transplant centers [124]. By recognizing and mitigating the biologic consequences of donor death, clinicians can not only expand the donor pool safely but also improve outcomes and quality of life for heart transplant recipients worldwide.

## Keypoints

- Donor cause of death influences allograft inflammation, injury, and early function, particularly with DBD-associated cytokine release and DCD-associated ischemia.
- DCD hearts carry a higher risk of PGD, but newer techniques like NRP are mitigating early injury.
- DBD donors show higher inflammatory and immunogenic profiles, potentially increasing rejection risk.
- CAV and chronic rejection may be more associated with DBD donors, though long-term DCD data are emerging.
- Heterotopic transplantation remains a niche but valuable approach in specific recipients, offering potential for recovery and parallel support.
- Blood type O recipients face longer wait times, making matching and donor strategy critical.
- Recipient evaluation should include medical, psychosocial, and immunological factors, including absolute/relative contraindications.
- New biomarkers and imaging techniques like AlloMap, cfDNA, and cardiac MRI are improving graft monitoring.
- Tailored immunosuppression and donor management protocols based on donor death mechanism could improve outcomes.
- Expanding the donor pool safely requires integrating technology, physiology, and individualized care pathways.
